# Every cloud has a silver lining: how abiotic stresses affect gene expression in plant-pathogen interactions

**DOI:** 10.1093/jxb/eraa531

**Published:** 2020-11-14

**Authors:** Marco Zarattini, Mahsa Farjad, Alban Launay, David Cannella, Marie-Christine Soulié, Giovanni Bernacchia, Mathilde Fagard

**Affiliations:** 1 Institut Jean-Pierre Bourgin, INRAE, AgroParisTech, Université Paris-Saclay, Versailles, France; 2 PhotoBioCatalysis Unit – Crop Production and Biostimulation Lab (CPBL), Interfaculty School of Bioengineers, Université Libre de Bruxelles (ULB), CP150, Avenue F.D. Roosevelt 50, Brussels, Belgium; 3 Department of Life Sciences and Biotechnology, University of Ferrara, Via Borsari 46, Ferrara, Italy; 4 Sorbonne Universités, UPMC Univ. Paris 06, UFR 927, 4 place Jussieu, Paris, France; 5 Université Paris-Sud, France

**Keywords:** Abiotic stress, crosstalk, defense, plant–pathogen interaction, transcriptome, virulence

## Abstract

Current environmental and climate changes are having a pronounced influence on the outcome of plant–pathogen interactions, further highlighting the fact that abiotic stresses strongly affect biotic interactions at various levels. For instance, physiological parameters such as plant architecture and tissue organization together with primary and specialized metabolism are affected by environmental constraints, and these combine to make an individual plant either a more or less suitable host for a given pathogen. In addition, abiotic stresses can affect the timely expression of plant defense and pathogen virulence. Indeed, several studies have shown that variations in temperature, and in water and mineral nutrient availability affect the expression of plant defense genes. The expression of virulence genes, known to be crucial for disease outbreak, is also affected by environmental conditions, potentially modifying existing pathosystems and paving the way for emerging pathogens. In this review, we summarize our current knowledge on the impact of abiotic stress on biotic interactions at the transcriptional level in both the plant and the pathogen side of the interaction. We also perform a metadata analysis of four different combinations of abiotic and biotic stresses, which identifies 197 common modulated genes with strong enrichment in Gene Ontology terms related to defense . We also describe the multistress-specific responses of selected defense-related genes.

## Teaser

We review the effects of major abiotic stresses on the expression of pathogen virulence and plant defense genes, and conduct a metadata analysis of four multistress transcriptomic datasets.

## Introduction

In crop production, non-optimal growth conditions, i.e. abiotic stress, and pathogens are two major factors that can negatively affect yield, potentially leading to huge losses. It has long been known that abiotic stresses affect plant disease, and these interactions can be very important. For example, in the case of the nitrogen-induced susceptibility of rice to the fungus *Magnaporthe oryzae* that follows nitrogen fertilization, which Vietnamese farmers have named Koe-imochi (Ballini *et al.*, 2013). Current knowledge indicates that plant–pathogen interactions are affected during abiotic stress via the following factors: (i) plant metabolism, and hence nutrient availability for the pathogen; (ii) plant cell viability; (iii) signaling (for a review see Kissoudis *et al.*, 2014); and (iv) the transcriptomic regulation of both the plant and the pathogen. We will briefly summarize the first two points in this Introduction, and then focus on the second two in the rest of this review.

Since pathogens need to find appropriate and sufficient nutrients when invading plant tissue, abiotic stress is likely to affect pathogen nutrition *in planta* (Lemaitre *et al.*, 2008; Singh *et al.*, 2019). In some cases the pathogens themselves manipulate plant primary metabolism to their advantage, as in the case of infection of susceptible tomatoes by the necrotrophic fungus *Botrytis cinerea*, which induces the expression of asparagine synthetase, leading to the accumulation of asparagine in the infected tissue (Seifi *et al.*, 2014). Modifications to plant metabolism will affect pathogens to a greater or lesser degree depending upon their life cycle. Thus, biotrophs are generally thought to be more dependent on the metabolism of their host than necrotrophs (Ah-Fong *et al.*, 2019). Understanding the precise impact on pathogen fitness *in planta* of the modifications to the accumulation of primary metabolites that are induced by abiotic stresses is a complex and rather overlooked field (reviewed in Fagard *et al.*, 2014).

Abiotic stress can affect cell viability, and this in turn can affect the outcome of plant–pathogen interactions in many ways depending on the pathogen life cycle. For example, nitrogen-limitation favors the onset of senescence (Lemaitre *et al.*, 2008), which is beneficial to some necrotrophic pathogens. However, such an effect on tissue senescence does not give the whole picture since some necrotrophic pathogens are more virulent in high-nitrogen conditions (Fagard *et al.*, 2014).

Plant–pathogen interactions have been well studied and many key molecular factors have been identified both on the plant and the pathogen side (Gust *et al.*, 2017). Upon perception of the pathogen by the plant through recognition of pathogen/microbe-associated molecular patterns (P/MAMPs) by pattern recognition receptors, the first layer of immunity, termed PAMP-triggered immunity (PTI), is activated. Adapted pathogens can overcome PTI by releasing protein effectors inside plant cells using a type 3 secreting system in the case of many gram-negative bacteria, or by secreting them in the apoplast in the case of fungi and oomycetes. In turn, plants that possess specific resistance genes of the NBS-LRR family can sense virulence effectors. This specific recognition triggers a powerful defense response termed effector-triggered immunity (ETI) (Deslandes and Rivas, 2012). Activation of both PTI and ETI involves signaling pathways that require MAPK-signaling, which are regulated by the major phytohormones salicylic acid (SA), jasmonic acid (JA), and ethylene (ET), and which in turn activate downstream responses via a large array of transcription factors (TFs). Generally, this defensive line culminates in a hypersensitive response at the site of infection, together with the synthesis of antimicrobial molecules such as phytoalexins and pathogenesis-related proteins (for review see Berens *et al.*, 2017). Despite the extensive literature on plant–pathogen interactions, unfortunately little is known about how plant defense is affected by abiotic stresses. Furthermore, to the best of our knowledge, no review has addressed the subject of activation of virulence gene signals *in planta* and their modulation under different environmental constraints.

In this review, we focus on how abiotic stresses affect the expression of plant defense at the transcriptomic level, together with the expression of pathogen virulence. We focus on three major abiotic stresses: drought, extreme temperatures, and nitrogen starvation and other mineral deficits.

## Temperature, hormones, and defense genes: multifaceted crosstalk

**Fig. 1.**
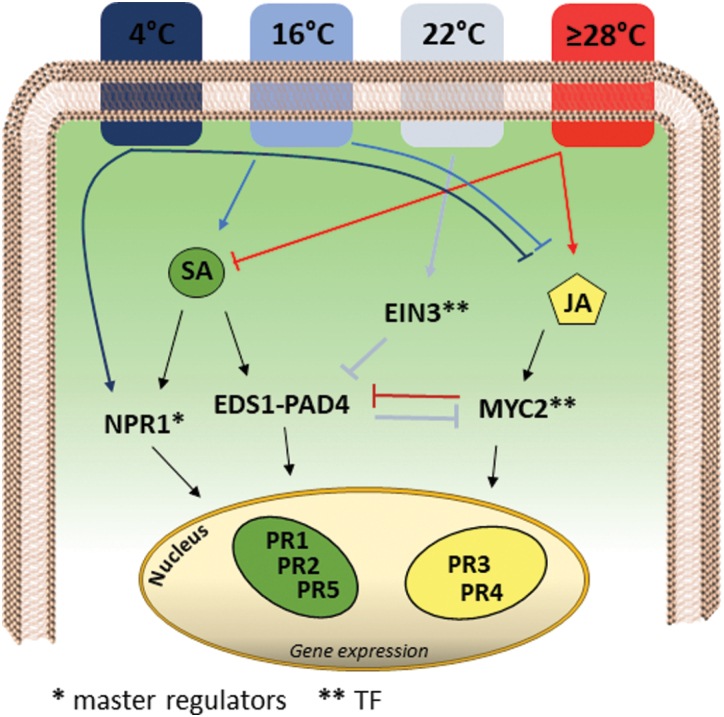
Modulation of plant-pathogen defense responses by cold and heat stress. Salicylic acid (SA), jasmonic acid (JA), and ethylene (ET) are the major phytohormones involved in plant–pathogen interactions and they are modulated differently by cold and heat stress. Plants exposed to low temperature show high levels of resistance, and several reports have indicated that cold stress results in SA-related defense being enhanced while JA-dependent signaling is inhibited. SA responses occur independently of SA accumulation and of the EDS1-PAD4 complex depending on the intensity of the cold treatment, whereas NPR1 is a significant factor in signaling cold-induced gene expression whether the intensity is mild or strong (Cui *et al.*, 2018; Olate *et al.*, 2018; Li *et al.*, 2020). An opposite scenario occurs at elevated temperature. SA biosynthesis is suppressed by the antagonist action of JA/ET. At 22 °C the transcription factor *EIN3* blocks SA-dependent defense (Li *et al.*, 2020) whilst the heat-induced JA responses may or may not be mediated via the*MYC2* transcription factor (Huot *et al.*, 2017; Mine *et al.*, 2017).

Under conditions favorable to growth, the activation of plant defenses is regulated by elaborate crosstalk among phytohormones such as SA, JA, and ET. In the present context of climate change, understanding how hormone-dependent gene expression is altered by ever-changing temperatures is of great interest. In the past few years, several reports have shown that the defensive responses mediated by SA during the interaction between Arabidopsis and *Pseudomonas syringae* are increased at low temperature (16 °C; Li *et al.*, 2020), and compromised under high (28 °C; Wang *et al.*, 2009) and extreme temperatures (37 °C and 42 °C; Janda *et al.*, 2019). Interestingly, extreme temperatures compromise defense even when the exposure is short (Janda *et al.*, 2019). An elegant study by Huot *et al.*, (2017) reported that at an elevated temperature of 30 °C, Arabidopsis plants exposed to the synthetic SA analogue benzothiadiazole did not accumulate mRNA of the two SA-marker genes *ICS1* and *PR1* (see Supplementary Table S1 for a list of all Arabidopsis genes mentioned in this review). Moreover, several other positive regulators of SA biosynthesis and signaling, namely *EDS1*, *PAD4*, *CBP60g*, and *SARD1*, were negatively affected by elevated temperature. Interestingly, the inactivation of SA-responsive genes at 30 °C was unrelated to the inability of NPR1 to translocate to the nucleus. Instead, it appeared that elevated temperature affected SA-dependent gene expression through the activation of negative SA regulators. For example, *MYC2*, a master regulator gene of JA signaling and a negative regulator of SA signaling, showed higher expression levels at 30 °C than at 22 °C (Huot *et al.*, 2017). Thus, at high temperatures, JA may confer susceptibility to *P. syringae* through negative regulation of *PAD4* that is mediated by *MYC2* and its homologs *MYC3* and *MYC4*. Therefore, it appears that high temperature conditions lead to the suppression of SA responses due to the antagonist effect of JA signaling (Fig. 1). On the other hand, SA signaling is also known to antagonize JA signaling.

A different scenario occurs when plants are grown at temperatures below their optimum. There is evidence indicating that cold stress confers increased disease resistance against hemi- and biotrophic pathogens. It has recently been reported that short-term cold stress at 4 °C positively modulates SA-dependent responses at the expense of the JA defensive pathway in Arabidopsis (Wu *et al.*, 2019). In particular, the SA-marker genes *PR2* and *PR5* are up-regulated by cold treatment whereas the JA markers *PR4* and *MYC2* are inhibited by cold. Similarly, SA-dependent activation of *PR1*, *PR2*, and *PR5* is observed in Arabidopsis exposed to long-term cold conditions (Seo *et al.*, 2010). Moreover, the SA-dependent responses appear to play a key role in increasing the resistance to *P. syringae* even at a moderately low temperature of 16 °C (Li *et al.*, 2020). By using transcriptomic and knock-out mutants for SA, JA, and ET signaling, the authors demonstrated that *PAD4* and *ICS1* are critical components of the SA-dependent responses in Arabidopsis at this temperature. On the other hand, the up-regulation of multiple SA-inducible genes, namely *PR1*, *PR2*, *EDS1*, *WRKY18*, and *WRKY30*, was shown to be negatively affected by *EIN3*, a master regulator of the ET-signaling pathway (Li *et al.*, 2020). Thus, higher resistance to *P. syringae* at low temperature relies on SA–ET crosstalk that results in up-regulation of SA-dependent responses.

Taken together, it appears that at elevated temperatures, JA-dependent responses down-regulate SA-dependent signaling, leading to increased susceptibility to *P. syringae*, whereas cold temperatures mainly boost the SA-dependent response, leading to increased resistance to *P. syringae* (Fig. 1).

Recent data have opened up a new perspective on these processes. Olate *et al.* (2018) have shown that *NPR1* can act as a hub in the molecular crosstalk between cold and biotic stresses, in an alternative regulatory mechanism to the canonical hormonal signaling network (Fig. 1). At low temperatures, NPR1 moves to the nucleus and regulates numerous genes associated with the responses to cold and pathogens (*PR2*, *WRKY46*, *DMR6*, *NAC019*) via interaction with the TF HSFA1 (Olate *et al.*, 2018).

## Temperature stress and virulence genes: the pathogen point of view

Plant pathogens usually only undergo gradual temperature changes during seasonal cycles and are generally not subjected to sudden temperature changes (Bocsanczy *et al.*, 2014). However, due to climate change, extreme temperature events are predicted to occur more rapidly and more frequently. Extreme temperatures can directly affect pathogen physiology in different ways, which in turn can influence the outcome of plant–pathogen interactions. Several studies have described the adaptation of pathogen physiology to low or moderate temperatures (Ramos *et al.*, 2001), including modifications of expression of virulence factors *in planta*, a key step in pathogenesis. However, the number of studies addressing the subject of virulence gene expression in plants grown at high temperatures is relatively low.

At low temperatures, a modification of virulence gene expression is observed for pathogens adapted to temperate climates. For example, *Erwinia amylovora*, the phytopathogenic bacterium responsible for fireblight in the Maloideae family, can adapt to lower temperatures (4 °C and 14 °C) by increasing the production of exopolysaccharides, which are involved in biofilm formation and in resistance to oxidative stress (Santander and Biosca, 2017). Another example of adaptation to low temperatures is found in another phytopathogenic bacterium *Ralstonia solanacearum*, a tropical pathogen, in which a few strains adapted to temperate climates do not show any reduction in their metabolism at a moderately low temperature of 18 °C (Bocsanczy *et al.*, 2014). Interestingly, the differences in virulence are primarily explained by changes in temperature-dependent gene expression of the virulence regulators *hrpB* and *hrpG*. In addition, this study highlighted a role in virulence of a putative type 6 secretion system not previously associated with infection (Bocsanczy *et al.*, 2014). Another study by Meng *et al.* (2015) focused on transcriptome responses in *R. solanacearum* to low temperature (4 °C) and showed an up-regulation of specific genes only in cold-adapted strains. Three of these genes (*LecM*, *AidA*, *AidC*) were required for full virulence, of which two (*LecM*, *AidC*) were present only in the genome of the adapted strains. Taken together, these studies point to a temperature-dependent regulation of virulence genes, whether they be known or novel, to explain the different virulence phenotypes observed at low temperatures.

High temperatures have also been shown to affect virulence in pathogens, and generally result in an increase. For example, translocation of *P. syringae* type III effectors increases at high temperature (Huot *et al.*, 2017). In rice plants challenged with the fungus *Magnaporthe oryzae*, which causes rice blast, stronger necrotic symptoms are observed at high temperatures (Onaga *et al.*, 2017), and transcriptome analysis has consistently indicated that many putative *M. oryzae* effector genes are more highly expressed in plants exposed to 35 °C than to 28 °C. A temperature rise could therefore increase the incidence and severity of rice blast, a serious threat that should not be underestimated in the present scenario of climate change (Onaga *et al.*, 2017). Similar results have also been observed in the bacterium *Dickeya solani*, an emerging pathogen responsible for soft rot and blackleg in potato crops. At high temperatures, *D. solani* causes more severe symptoms than other *Dickeya* species, suggesting a temperature-dependent boost of virulence in adapted strains and species (Czajkowski *et al.*, 2016). High temperatures have been shown to result in the up-regulation of 45 *D. solani* genes, four of which are required for biofilm production and virulence in potato. Interestingly, these key genes do not encode cell wall-degrading enzymes but a putative phospholipase (*plcA*), rhamnogalacturonase (*rhiN*), lysine aminomutase (*yodO*), and a regulatory protein (*araC*). The up-regulation of these loci could play a key role in the fitness of *D. solani* at high temperatures, a bad omen for potato crops given current climate change.

Some studies have reported a negative regulation of virulence under higher temperatures. A recent study by Saha *et al.* (2015) examined the effects of an array of temperatures between 18–37 °C on the phytopathogenic bacterium *Pectobacterium carotovorum*, which is responsible for bacterial soft rot in a wide range of plant species. The authors identified an optimal temperature of 33 °C for the production of the quorum-sensing signal molecule, acyl homoserine lactone, which regulates the bacterial population and virulence gene expression. Beyond this optimum, no accumulation of the quorum-sensing molecule and no disease were observed. A second example of a negative impact of high temperatures on virulence factors can be seen in *P. syringae*, in which the production of the phytotoxin coronatine is repressed at 28 °C compared to 18 °C (Ullrich *et al.*, 1995).

Overall, most studies have found that high temperatures tend to favor pathogen virulence, while low temperatures tend to decrease virulence except in adapted strains. However, increasing temperatures beyond the optimal level for the expression of pathogen virulence will most likely decrease virulence, as seen in the example *P. carotovorum*.

## Water stress: a positive or negative regulator of plant defense genes?

Drought stress is another major environmental factor that affects plant physiology, metabolism, and growth, and its occurrence is becoming increasingly worrying in many parts of the world. It can be caused by several phenomena, such as dehydration, salinity, high or low temperatures, and its effects depend on timing, severity, and the presence and types of interactions with other factors (Salehi-Lisar and Bakhshayeshan-Agdam, 2016). The whole plant defense system can be expected to be affected by water stress, but interestingly drought has been shown to cause both detrimental and beneficial effects on plant–pathogen interactions, both in terms of resistance and gene expression. Accumulation of abscisic acid (ABA) is very often observed in plants exposed to drought, and this leads to stomatal closure that prevents bacteria from entering through stomata, and also to other physiological responses with a putative role in plant–pathogen interactions (Melotto *et al.*, 2017; Zarattini and Forlani, 2017). However, the precise role of ABA in plant–pathogen interactions is still a matter of debate. ABA can interact either synergistically or antagonistically with other defensive hormones such as SA, JA, and ET, thus affecting the outcome of biotic stress (reviewed in Cao *et al.*, 2011).

As might be expected, there are frequent examples of plants being more susceptible to pathogens after a period of drought. For example, rice exposed to moderate drought conditions show higher susceptibility to *M. oryzae* (Bidzinski *et al.*, 2016), which is due to lower expression of the defense marker genes *PAL*, *PBZ1*, *POX22.3,* and *PR3*. Water stress also appears to inhibit the immune system in forest trees. Transcriptomic and metabolomic analyses of seedlings of pine (*Pinus koraiensis*) challenged with *Cenangium ferruginosum* after experiencing water stress show that expression of defense genes is impaired (Ryu *et al.*, 2018). In addition, reduced synthesis of specialized metabolites such as terpenoids, flavonoids, and phenolic acids is also observed whereas the levels of ABA are increased.

Drought can also have a positive effect on defense. For example, drought-stressed Arabidopsis and chickpea show enhanced resistance to the bacterial pathogens *P. syringae* DC3000 and *P. syringae* pv. *phaseolicola*, respectively (Gupta *et al.*, 2016; Sinha *et al.*, 2017). Drought-acclimated *Nicotiana benthamiana* plants show higher accumulation of mRNA of *PR5* and *PDF1.2* that leads to enhanced resistance to the fungus *Sclerotinia sclerotiorum* and the bacterium *P. syringae* pv. *tabaci* (Ramegowda *et al.*, 2013). Cramer *et al.* (2006) found that drought increases the expression of the defense-related genes *PR*5, *PR2* and *Germin-like1.15* in grapevine. Comprehensive RNA-seq analysis of 2-year-old plants subjected to drought stress revealed that 72 genes encoding pathogenesis-related proteins were differentially expressed following drought (Haider *et al.*, 2017); in particular, transcripts of several *PR* genes (*PR1*, *PR2*, *PR3*, *PR5*, *PR10*, *PR14*, and *PR15*) were positively modulated. Other studies have shown that application of PEG, sucrose, or salt to mimic the osmotic stress induced by drought can induce defense (Hatmi *et al.*, 2014; Guan *et al.*, 2018). For example, among the 35 *WRKY* genes induced by *Fusarium udum* infection in pigeonpea (*Cajanus cajan*), 11 were also induced by salt stress (Kumar *et al.*, 2019).

Predicting *a priori* the impact of drought (as well as osmotic) stress on plant–pathogen interactions and plant defense therefore appears to be particularly difficult. Reduced water availability usually negatively affects plant physiology and growth; however, this is not always true when plants face a pathogen attack. The actual disease outcome is strongly dependent on the pathosystem that is being considered, and hence on how signaling pathways triggered by water and biotic stress interact to affect the expression of defense genes.

## How pathogens respond to drought: is it possible to maintain or even increase virulence?

As outlined above, drought stress strongly affects plant defense but also induces various metabolic and physiological changes in the plant tissues. Phytopathogens that attack aerial organs often deal with the various stresses that they encounter on the surface of the host leaves by accumulating compatible osmolytes, and this includes the response to water limitation during the epiphytic phase (Bremer and Krämer, 2019). Such osmotic stress conditions not only interfere with the general metabolism and life cycle of phytopathogens but can also affect their pathogenic cycle. Thus, the pathogen’s capacity to cope with water limitation both in the phyllosphere and inside the leaf tissue will affect disease progression. Several studies have shown that salt stress, which also causes osmotic stress, can alter the expression of virulence genes. Although the question of whether plant pathogens alter their virulence when faced with water stress has not been extensively studied, several examples of induction of virulence genes by water limitation have been described.

The bacterial pathogen *Xanthomonas citri* subsp. *citri* spends part of its life cycle on the surface of citrus leaves, where it can face water limitation. By applying saline stress to mimic water limitation, Barcarolo *et al.* (2019) have identified proteins involved in bacterial tolerance to reactive oxygen species (ROS) that accumulate *in vitro*, including a putative NADPH dehydrogenase. Expression of the corresponding gene, *Xac2229*, is induced both by saline stress *in vitro* and during the plant–pathogen interaction. Interestingly, *Xac2229* is required for virulence *in planta* but does not confer any advantage to *X. citri* under salt stress *in vitro*. This suggests that *Xac2229* could be required for bacteria to cope with indirect effects of water limitation *in planta* rather than with water limitation/salt stress *per se*. Another study has examined *Alternaria brassicicola*, a necrotrophic fungus that causes important damage to cultivated Brassicaceae. Seed transmission is an important part of the life cycle of this fungus and this requires that the it can resist low water availability (N’Guyen *et al.*, 2019). An *in vitro* transcriptome analysis of the fungus under water-limiting conditions resulted in the identification of a group of hydrophilin-encoding genes that show transcriptional activation under water limitation. Analysis of the corresponding knock-out mutants indicated that the genes are not involved in fungal virulence in plants grown in optimal conditions but that they are required for full transmission of the pathogen spores by Arabidopsis seeds (N’Guyen *et al.*, 2019). Although these mutants show wild-type virulence under control conditions, whether the identified genes play a role in virulence in plants subject to water limitation remains an unanswered question.

Drought stress has also been shown to increase the aggressiveness of the fungus *M. oryzae*. RNA-seq analysis performed on fungal hyphae *in planta* revealed differences in the fungal gene expression profile between well-watered plants and plants exposed to drought conditions (Bidzinski *et al.*, 2016). In particular, drought reduces the *in planta* expression of genes encoding effectors, such as *Avr*-*PITA*, and induces genes encoding cell wall-degrading enzymes. These results suggest that the fungus adapts its virulence program to the physiology of the stressed plants, probably through unknown signals produced by the plant and perceived by the fungus.

Although the literature on the subject remains limited, the current available data suggest that increases in the occurrence of drought stress will not only affect plants but will also increase the capacity of pathogens to express virulence through as yet unknown mechanisms.

## Nutrient limitation: emerging implications for plant defense gene expression

Mineral depletion causes stress for plants and affects many different processes including defense. Several recent studies have described how mineral depletion affects the expression of genes associated with biotic stress. For example, genes involved in JA biosynthesis and signaling in barley (*HvLOX2A*, *HvAOC*, *HvJIP60*, *HvJIP23*, *HvJIP37*, and *HvAOS*) are induced by low potassium supply (Davis *et al.*, 2018). Interestingly, this is correlated with an increase in resistance to *Blumeria graminis*, a JA-susceptible pathogen causing powdery mildew. In Arabidopsis, potassium deficiency induces the expression of JA-dependent downstream genes (Armengaud *et al.*, 2010). These observations suggest that alongside the triggering of responses related to potassium depletion, such as up-regulation of the high-affinity K transporter *HAK5*) (Armengaud *et al.*, 2004), plants also activate hormone-dependent defensive responses that are able to increase pathogen resistance. Surprisingly, opposite results have been observed in rice seedlings grown with low potassium, with decreased expression of two JA-dependent genes, *OsLOX5* and *Os12g14440*, being observed (Shankar *et al.*, 2013). However, it is currently difficult to know whether these differences are species-specific (rice versus barley and Arabidopsis) or whether they are due to differences in the experimental set-ups.

**Fig. 2.**
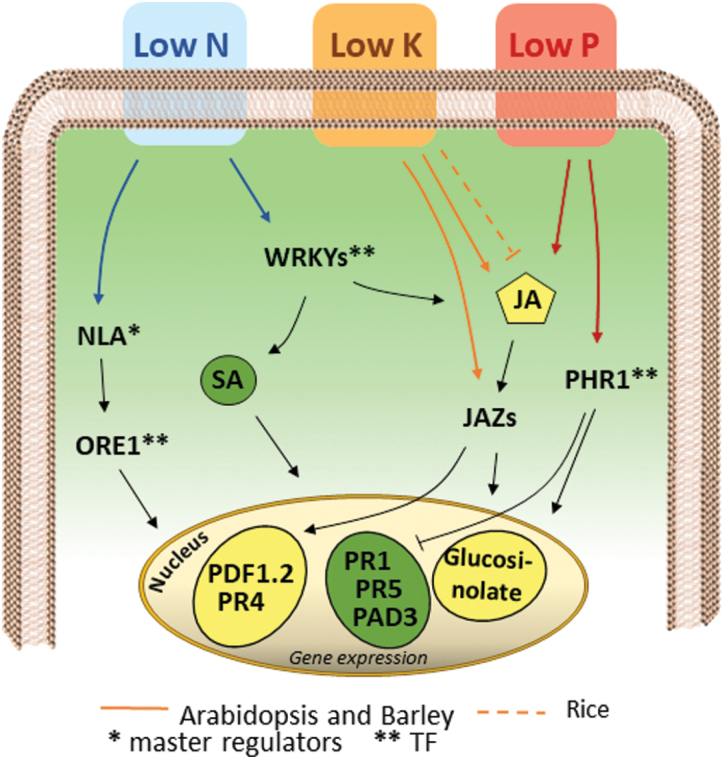
Modulation of plant-pathogen defense responses by mineral limitation. Increasing evidence indicates that nutritional status has an impact on plant defense. In Arabidopsis, phosphate limitation can modulate defense signaling either via the jasmonic acid (JA) pathway or via PHR1, a master regulator that governs responses to phosphate starvation. PHR1 has a dual function of modulating the plant immune system, either by inhibiting the expression of salicylic acid (SA)-dependent genes or by activating a subset of JA-responsive genes that are mainly involved in glucosinolate biosynthesis (Castrillo *et al.*, 2017). Low potassium leads to opposite responses in Arabidopsis, barley, and rice (Armengaud *et al.*, 2010; Shankar *et al.*, 2013). Although low potassium leads to an increased level of JA and expression of JAZs genes in Arabidopsis and barley, a decreased level of JA occurs in rice. Upon low nitrate conditions, genes belonging to the WRKY transcription factor family are induced in Arabidopsis (Patterson *et al.*, 2010), which in turn can regulate the SA/JA balance as well as hormone-related gene expression. Interestingly, a direct interaction between NLA and ORE1, two key regulators of nitrogen limitation and senescence, has recently been demonstrated. ORE1 is a NAC transcription factor (NAC092) that might modulate JA-dependent gene expression (Park *et al.*, 2018).

Phosphate and nitrogen limitations have also been shown to affect the expression of plant defense genes. Transcriptome analysis of roots of *Medicago truncatula* grown with a combination of low phosphate and low nitrogen has indicated induced expression of several stress-associated genes, including ones encoding NADPH oxidases (Bonneau *et al.*, 2013). A recent study by Castrillo *et al.* (2017) in Arabidopsis examined the link between regulation of the immune system and the formation of microbiota under phosphate starvation. The authors concluded that the master regulator of phosphate starvation, *PHR1*, down-regulates SA-dependent responses while increasing the expression of JA-associated genes, mostly those related to glucosinolate biosynthesis (Fig. 2).

Plants can use nitrogen in both oxidized and reduced forms, mainly as nitrate and ammonium. Arabidopsis roots grown in low nitrate or low ammonium show both common responses (a generic nitrogen-limitation response) as well as specific responses to the limitation in each nitrogen source (Patterson *et al.*, 2010). In particular, this study showed that low ammonium triggered the expression of biotic-associated genes such as *WRKY70*, which regulates the SA/JA balance, and JA-responsive genes (Fig. 2). Another study has shown that growth in ammonium reduces resistance to an avirulent strain of *P. syringae* due to lower production of NO (Gupta *et al.*, 2012). Ammonium triggered the accumulation of specialized metabolites, suggesting that nitrogen availability not only affects mineral homeostasis in the plant cells but can also activate defense at both the molecular and the biochemical levels.

**Fig. 3.**
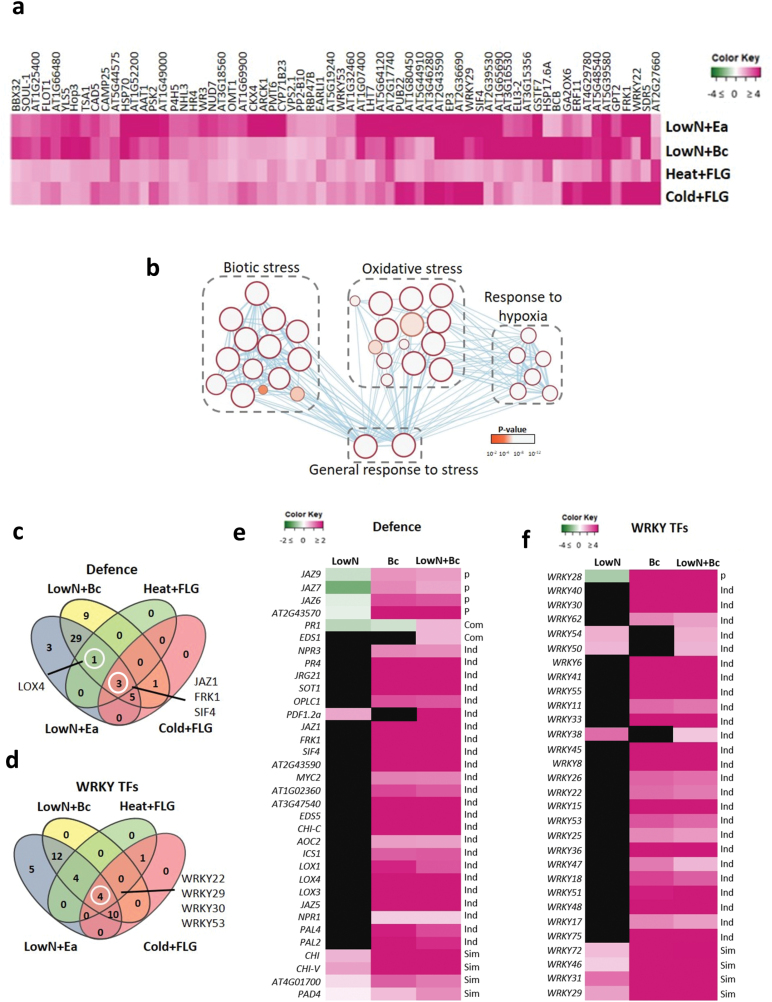
Meta-analysis of combined abiotic and biotic stress transcriptome data. Ten publicly available datasets were selected to study the modulation of defense gene expression in response to combined stresses. Transcriptome data for nitrate limitation (LowN), *Botrytis cinerea* (Bc), and their combination were obtained from Soulié *et al.* (2020), whilst the data for cold and flagellin (FLG) and for heat and FLG were extracted from the Gene Expression Omnibus repository (accession GSE41935; Rasmussen *et al.*, 2013). (a) Expression of 66 commonly up-regulated genes in all four multistress conditions (fold change >1; see Supplementary Table S2). (b) Gene Ontology analysis was performed using the Cytoscape and g:Profiler software according to Reimand *et al.* (2019) using the total of 197 up- and down-regulated genes shared in all multistress conditions (fold-change >1 or <–1; Supplementary Table S2). (c. d) Venn diagrams showing (c) three defense genes and (d) four WRKYs commonly modulated by all the combined stress conditions. (e, f) Heat-maps showing expression of selected defense genes (e) and WRKY transcription factors (d), following single LowN stress, *Bc* infection, and their combinations. Black shading indicates genes not significantly modulated (*P*>0.05; Supplementary Tables S2, S3). The same genes reported in the heat-maps were also screened in datasets related to the combination of heat, cold, and flagellin (see Supplementary Table S3).

Switching the nitrogen source to nitrate appears to have contrasting effects depending on the plant species considered. In tomato plants exposed to low nitrate, a principal component analysis showed that the transcriptional response clustered close to that of plants infected by *Botrytis*, strongly suggesting that low nitrate primes defense responses even in the absence of infection (Vega *et al.*, 2015). Our own studies have shown that nitrogen limitation also affects the activation of transcriptional defense in Arabidopsis leaves in responses to bacterial infection (Farjad *et al.*, 2018), fungal infection (Soulié *et al.*, 2020), and to defense stimulators (Zarattini *et al.*, 2017; Verly *et al.*, 2020). In the absence of a pathogen or defense stimulator, several WRKY TFs are positively modulated when the nitrate source is limited, even if a weaker magnitude of expression is generally observed as compared to plants infected with pathogens (Fig. 3). Moreover, nitrate limitation alters the defense responses triggered by pathogens and defense stimulators. For example, in Arabidopsis low nitrate boosts the induction of *PDF1.2a* by the defense stimulator deoxycholic acid, a bile acid (Zarattini *et al.*, 2017), and by *B. cinerea*, thus increasing resistance to this fungus (Soulié *et al.*, 2020). Taken together, our data indicate that nitrate limitation strongly affects defense signaling pathways in response to a variety of biotic stimuli, emphasizing the importance of JA signaling in the integration of nutritional and defense cues.

Mineral depletion represents a serious threat to agriculture since it affects plant growth and is a major cause of yield loss in crops. Molecular and transcriptomic studies indicate that stress linked to mineral depletion often primes defense responses; however, negative effects of mineral depletion on defense have been reported for multiple pathosystems. This should be taken into consideration for each crop when selecting cultivars and fertilization.

## When nutrient limitation reaches the pathogen: a signal for virulence genes

Soil mineral depletion affects both plant metabolism and the chemistry of root and leaf exudates, which in turn affect the interactions of plants with their surrounding microbes, beneficial or not. Many studies have shown that plant growth conditions, in particular nitrogen availability, alter the capacity of nitrogen-fixing bacteria to establish symbiosis and alter the transcriptome of plant growth-promoting rhizobacteria in the soil (Carvalhais *et al.*, 2013). However, little data exist concerning the effects of plant nutrient limitation on pathogen virulence. Many pathogens express their virulence factors specifically when infecting plants and not when grown in rich medium *in vitro* ([Bibr CIT0060][Bibr CIT0073]). However, relatively few studies have addressed the actual metabolic environment encountered by pathogens *in planta*, and the signals that allow the expression of pathogen virulence genes *in planta* are not well known yet. On the other hand, several studies have shown that pathogens express their virulence factors *in vitro* when grown in limiting nutrient conditions (Bolton and Thomma, 2008; Tudzynski, 2014). For example, low nitrogen and low carbon both induce the *Magnaporthe grisea* gene *Mgp1*, and low nitrogen induces the *avr9* gene in *Cladosporium fulvum* (Talbot *et al.*, 1993; Van den Ackerveken *et al.*, 1994). In *Fusarium oxysporum*, production of fusaric acid, a toxin required for disease, is greater *in vitro* with high nitrate (5 mM) than with low nitrate (1 mM) or ammonium (Zhou *et al.*, 2017); the pathogen induces stronger disease symptoms when infecting plants grown with ammonium than with nitrate, indicating that there is no strict correlation between what is observed *in vitro* and *in planta*.

Taken together, *in vitro* studies have led to the hypothesis that nutrient limitation could represent a signal for the induction of virulence genes; however, *in planta* data to support this remain scarce and mostly indirect (Wilson *et al.*, 2012). For example, it is well known that fungal secondary metabolism is affected by nitrogen sources, as shown for *F. oxysporum* (Tudzynski, 2014; Sharma and Jha, 2015). In *Ustilago maidis*, the Nit2 TF, which activates the fungal nitrogen catabolite repression process, has also been shown to regulate virulence since the *nit2* mutant possesses reduced virulence (Horst *et al.*, 2012). This would indicate that the source of nitrogen and its metabolic pathway not only modulates the pathogen biology but also its virulence, an aspect worth exploring to improve plant tolerance to biotic stresses.

Some rare studies have directly analysed the expression of virulence factors in plants grown with contrasting levels of fertilization. For example, *M. oryzae* expresses high levels of pathogenicity-related and effector genes in host plants grown under high nitrogen regimes (Huang *et al.*, 2017). We have analysed virulence factors of *E. amylovora* and *B. cinerea* in plants grown on low or high nitrate and found that stronger symptoms are associated with higher expression of virulence factors and pathogenicity-related genes, which are observed under low nitrate for *E. amylovora* and under high nitrate for *B. cinerea* (Soulié *et al.*, 2020; M. Farjad *et al.*, unpublished results). Interestingly, among the highest expressed *B. cinerea* genes in high nitrate, we demonstrated for the first time the involvement in virulence of two genes that encode a protease (*acp1*) and a secondary metabolite biosynthesis enzyme (*sm*). *SM* encodes a putative oxydoreductase orthologous to the *Cochliobolus heterostrophus* gene *RED1* that is involved in the synthesis of T-toxin. In addition, a third gene corresponds to the well-known *bot2* that is involved in the biosynthesis of the toxin botrydial (Soulié *et al.*, 2020).

Limitation of inorganic phosphate (Pi) can also be encountered by bacterial pathogens in the soil or *in planta*. Bacteria perceive Pi deficiency through the two-component PhoBR signal transduction system, which leads to activation of the Pho regulon, allowing Pi uptake and assimilation (Chekabab *et al.*, 2014). Interestingly, several studies have shown that PhoBR also regulates bacterial virulence. This has mostly been studied in animal pathogens, but a few studies concerning phytopathogens exist (Petters *et al.*, 2002). For example, in *Agrobacterium tumefaciens*, PhoB is essential for virulence, and low-Pi conditions induce biofilm formation and catalase-encoding genes that protect bacteria against oxidative stress (Mantis and Winans, 1993; Chekabab *et al.*, 2014). In *Xanthomonas oryzae*, the pathogen of rice bacterial leaf blight, a PhoR loss-of-function mutant shows strongly reduced virulence (Zheng *et al.*, 2018). Transcriptome analysis of this *ΔphoR* mutant shows that several *hrp* genes that are required for the synthesis of the type 3 secretion apparatus and effector proteins are down-regulated. However, this study also showed that the PhoBR regulon was not activated *in planta*, suggesting that the bacteria encountered Pi-rich conditions and that the main role of PhoBR could be during the nutrient-poor epiphytic stages of the bacterial life cycle.

Taken together, our current knowledge suggests that nutrient availability for plants affects the transcription of pathogenesis-related genes during infection; however, these effects seem to be pathogen-dependent and probably plant-pathogen dependent as well. Although this remains to be studied, it is likely that signals perceived by pathogens *in planta* are affected by plant metabolism, possibly in the form of secondary metabolites, which are themselves linked to mineral nutrition conditions.

## Multistress signals orchestrate plant transcriptomic responses

In the past, most transcriptomic studies of abiotic and biotic stresses have examined them individually, a situation that rarely occurs under natural conditions. Analysis of data acquired in recent years, however, has led to the conclusion that abiotic and biotic stresses not only often occur simultaneously, but that the corresponding regulatory pathways can interact at several levels inside the plant. Recently, researchers have started to examine the transcriptomic responses of plants challenged with both biotic and abiotic stress (Table 1). Although the number of datasets remains limited, some lessons can be learned from their analysis. The first is that a very large number of genes responsive to combined stresses cannot be predicted from their responses to each single stress (Rasmussen *et al.*, 2013; Farjad *et al.*, 2018). The number of these genes that show a specific and non-predictable response to combined stresses varies from ~30% to ~60% of modulated genes depending both on the nature and the intensity of the combined stresses. These non-predictable genes show either a ‘prioritized’, ‘cancelled’, or ‘combinatorial’ response to stress combinations (as described below). Secondly, only a small percentage of genes are similarly modulated in their responses to numerous stress conditions, whether individual or combined (Prasch and Sonnewald, 2013). Thirdly, genotype plays an important role in the way plants integrate multistress signals. For example, high temperatures decrease the resistance to *X. oryzae* of rice carrying *Xa4* resistance but increase resistance of rice carrying *Xa7* resistance*, and* this is correlated with genotype-specific transcriptomic profiles under the multistress combination (Table 1; Dossa *et al.*, 2020). The importance of genotype is supported by the involvement of *PBS3*, an actor in SA signaling, in the age-dependent trade-off between two abiotic stresses and immune responses in Arabidopsis (Berens *et al.*, 2019): immune responses are reduced by drought and high salinity in older leaves, but not in younger leaves in which *PBS3* antagonizes the trade-off. Finally, in a multistress combination one stress can outweigh another (Coolen *et al.*, 2016; Davila Olivas *et al.*, 2016). In particular, the response to sequential application of stresses most resembles the response to the last-occurring stress, although a signature of the first stress is present.

**Table 1.** Summary of main conclusions drawn by studies analysing transcriptomic responses of plants to different abiotic–biotic multistress combinations

Stress combination	Plant species	Key conclusions	Reference
**Low N**			
*Botrytis cinerea*	*Solanum lycopersicum*	Nitrate limitation activates JA signaling and represses SA signaling in response to *Bc*	Vega *et al.* (2015)
*B. cinerea*	*Arabidopsis thaliana*	Nitrate limitation activates JA signaling and represses SA signaling in response to *Bc*; 182 *A. thaliana* and 22 *B. cinerea* genes specifically modulated by stress combination	Soulié *et al.* (2020)
*Erwinia amylovora*	*A. thaliana*	~30% of modulated genes show a specific response to stress combination	Farjad *et al.* (2018)
**Cold**			
Flagellin (*flg22*)	*A. thaliana*	~50% of modulated genes show a specific response to stress combination	Rasmussen *et al.* (2013)
**Drought**			
*Pseudomonas syringae*	*A. thaliana*	~30% of modulated genes show a specific response to stress combination, among which 150 remain specifically modulated independently of the order of stress application	Gupta *et al.* (2016)
*Magnaporthe oryzae*	*Oryza sativa*	Strong modification of fungal virulence program by drought: repression of small secreted proteins, activation of cell wall-degrading enzymes. Repression of effector-triggered immunity under drought.	Bidzinski *et al.* (2016)
*Botrytis cinerea*	*A. thaliana*	Second stress is dominant in transcriptome response but contains the first-stress signature	Coolen *et al.* (2016)
**High temperature**			
*flg22*	*A. thaliana*	~50% of modulated genes show a specific response to stress combination	Rasmussen *et al.* (2013)
SA-analog (BTH)	*A. thaliana*	Down-regulation of SA pathway	Huot *et al.* (2017)
*flg22*	*A. thaliana*	~50% of modulated genes show a specific response to stress combination	Rasmussen *et al.* (2013)
*Xanthomonas oryzae*	*O. sativa*	Up-regulation of ABA biosynthesis genes and down-regulation of SA pathway	Cohen *et al.* (2017)
*X. oryzae*	*O. sativa*	Down-regulation of cell wall biosynthesis genes in susceptible line; up-regulation of trehalose biosynthesis gene in resistant line	Dossa *et al.* (2020)
**Drought × High temperature**			
*Turnip mosaic virus*	*A. thaliana*	23 genes specifically modulated by triple stress combination; 11 genes modulated in all three stress conditions	Prasch and Sonnewald (2013)

To further decipher the impact of different stress combinations, we selected 10 transcriptomic datasets for analysis (Supplementary Table S2). These comprise single cold, heat, and flagellin treatment and their combinations, together with single low-nitrate, *E. amylovora*, and *B. cinerea* stress and their combinations (Rasmussen *et al.*, 2013; Farjad *et al.*, 2018; Soulié *et al.*, 2020). When comparing all 10 datasets, we identified only four genes that were significantly modulated among all single and combined stress conditions, which is consistent with previous observations made on other multistress combinations (Prasch and Sonnewald, 2013). Interestingly, these stress-robust genes comprise a putative kinase, a membrane glycoprotein, and a putative TIR-domain NBS-LRR resistance protein, none of which has yet been functionally characterized (Supplementary Table S2). We then focused on the four stress combinations and identified 197 genes that were modulated in all of them (Fig. 3a, Supplementary Table S2). Interestingly, these included several members of the WRKY and NAC TF families together with the defense-signaling kinase *FRK1* and the leucine-rich repeat receptor-like kinase *SIF4*. Gene Ontology analysis performed on all 197 genes highlighted a strong enrichment in the defense-related terms ‘response to biotic stress’ (14 nodes), ‘oxidative stress’ (14 nodes), and ‘response to hypoxia’ (six nodes) (Fig. 3b). Taken together, these data indicate that multistress-robust responsive genes are not found among the genes that respond specifically to multistress (i.e. and not to single stresses) but among the genes that also respond to some single stresses and remain activated in response to a variety of multistress combinations. These multistress-robust genes can be considered to be generally stress-robust and are of great interest for future research.

In order to better understand the modulation of expression of defense genes in response to stress combinations, we checked the expression of a manually curated list of genes (~1300) covering different aspects of plant defense in our multistress transcriptomic data (Supplementary Table S3). This list contains genes related to defense as well as the family of WRKY TFs, which are known to play a key role in the response to both biotic and abiotic stresses. We first compared the modulation of these genes in the 10 datasets of our analysis and found three defense-related genes (Fig. 3c) and four WRKY TFs (Fig. 3d) to be modulated by all stress combinations, suggesting that they are robust stress-response genes. We then used previously defined categories depending on whether their response to stress combinations could be predicted from their response to both single stresses (independent and similar categories) or not (prioritized, combinatorial, and cancelled categories), thus revealing an interaction between the response to the stresses (Rasmussen *et al.*, 2013; Farjad *et al.*, 2018). The response of most *WRKY* TFs genes was simply additive, with the response to one or both stresses being maintained (Fig. 3f: independent and similar responses, respectively). However, *WRKY28* showed a specific response to the multistress combination, with a prioritization of the response to *B. cinerea* over the response to nitrate limitation. The response of many defense-related genes to *B. cinerea* was maintained under nitrate limitation (Fig. 3e), as previously described (Farjad *et al.*, 2018). Interestingly, for several genes related to JA signaling (*JAZ 6*, *7*, *9*), the response to *B. cinerea* was prioritized over the response to nitrate limitation, while two genes related to SA signaling (*EDS1*, *PR1*) showed an induction specifically in the multistress combination (Fig. 3e). Our analysis thus shows that the defense response to *B. cinerea* overtakes the response to an abiotic stress, in this case nitrate limitation. This is opposite to the effect of heat, which had a negative effect on resistance and for which several defense and WRKY genes were found to be cancelled (Supplementary Table S3). These differences are consistent with a previous meta-analysis that showed that each multistress combination generated a specific response (Zandalinas *et al.*, 2019).

Taken together, analysis of multistress transcriptomic data point to a pivotal role in phytohormone signaling pathways in fine-tuning the plant’s response to multiple stresses. In our analysis, several genes involved the JA- and SA-dependent pathways showed a non-predictable response to multistress. Furthermore, several genes involved in phytohormone signaling were present in the first-stress signature in the response to the combined sequential stresses (Coolen *et al.*, 2016), again highlighting the key role of phytohormones as general integrators of multistress responses.

## Conclusions

Plants are constantly under the threat of both biotic and abiotic stresses. In this review, we have examined the literature to better understand how abiotic stresses affect the response of plants to biotic stress. This is of key importance, because abiotic stresses can have an impact early in the infection process as well as having implications for either the chemical and biological treatments used to prevent disease or the efficiency of genetic-based resistance.

The first way in which abiotic stress interacts with biotic stress is by directly activating or repressing genes that are known to be involved in responses to pathogens. Cold temperatures tend to repress JA-dependent genes and activate SA-dependent genes while high temperatures do the opposite (Fig. 1). Although genes associated with defense are generally modulated by abiotic stress at much lower levels compared to pathogen infections, their modulation by abiotic stress might affect the level of activation during a potential subsequent pathogen attack. Thus, a clear understanding of how abiotic stress impacts on the susceptibility of plants to pathogens is necessary and will require further investigation.

The second way by which abiotic stress affects biotic stress responses is by interfering with the signaling. The signaling crosstalk between biotic and abiotic stresses has been extensively described in the literature and we have not covered this aspect in the present review. In general, hormone signaling plays a key role in the integration of multistress signals. For example, the fine-tuning regulation of the SA/JA balance seems to be implicated in the integration of abiotic–biotic multistresses involving temperature and nutritional limitations. On the other hand, recent data indicate that the signaling of the abiotic-driven expression of defense genes might even occur independently of the accumulation of hormones, as in the response to cold, suggesting the existence of alternative signaling mechanisms (Fig. 1; Olate *et al.*, 2018). Besides hormones, TFs are key regulatory elements governing different aspects of multistress signals. For example, PHR1, a TF that regulates responses to phosphate starvation, has been demonstrated to repress SA-dependent genes and to activate JA-dependent genes (Fig. 2; Castrillo *et al.*, 2017). Metadata analysis, genome-wide TF-binding assays, and *in silico* modeling combined with technological advances, such as CRISPR systems, are examples of techniques that can potentially help to identify new regulatory genes implicated in the response of plants to multistress (Lai *et al.*, 2018). Hence, although great advances have been made in recent decades, further analysis will be required to get a clearer picture of the gene regulatory network that occurs during combined biotic and abiotic stress conditions. Furthermore, increasing the number of studies will allow a better comparison of datasets by meta-analysis. Our metadata analysis allowed us to identify a list of stress-robust genes that could be of great interest for future research, among which were several WRKY TFs and important defense-signaling genes (*FRK1*, *JAZ1*, and *SIF4*; Fig. 3). Our analysis also confirmed that the defense response can overtake the response to nutritional limitation.

The third way by which abiotic stress impacts on biotic stress is by affecting pathogen fitness and virulence inside the host plant. Once inside the leaf tissue, the pathogen is completely dependent on plant metabolism to perform its life cycle. Most studies nowadays on pathogen development *in planta* are performed on plants grown in optimal conditions; however, several recent studies have shown that studying pathogen virulence in non-optimal conditions can unveil novel virulence genes that are not evident in optimal conditions (Barcarolo *et al.*, 2020; Soulié *et al.*, 2020). This suggests that pathogens can adapt to variations in the physiology and metabolism of their host plant, which is consistent with the ability of some pathogen species such as *B. cinerea* to adapt to an array of important hosts (Blanco-Ulate *et al.*, 2014). Why do pathogens have virulence genes specifically expressed in plants undergoing abiotic stress? Further investigation is required to determine whether these virulence genes are unnecessary under optimal conditions or whether they allow adaptation to plants undergoing abiotic stress. However, the existing data should encourage us to look more closely into these conditions to identify new molecular actors and perhaps to understand part of the adaptability of pathogens. Finally, the data available, even though limited, suggests that pathogens perceive different plant signals in the leaf. Although studies of leaf pathogens are complicated since the analysis of leaf intercellular fluid is technically challenging, the current knowledge that we have suggests that an interesting development in the field of abiotic–biotic interactions would be to focus more on the plant-to-pathogen signaling that occurs *in planta*.

## Supplementary data

The following supplementary data are available at *JXB* online.

Table S1. List of Arabidopsis genes mentioned in the review.

Table S2. Complete datasets used for the metadata analysis, and lists of genes common to all stress conditions or common to all multistress conditions.

Table S3. Heatmaps and multistress profiles of selected defense-related genes and WRKY TFs.

eraa531_suppl_Supplementary_Table_S1

eraa531_suppl_Supplementary_Table_S2_S3

## Data Availability

The data that support the findings of this study are openly available in the Gene Expression Omnibus (GEO) repository, accessions GSE41935, GSE116135, and GSE97582.
