# Supplementary material for: Every cloud has a silver lining: how abiotic stresses affect gene expression in plant-pathogen interactions
Source: J Exp Bot. 2020 Nov 14;72(4):1020–33. doi: 10.1093/jxb/eraa531 (PMC7904152; doi:10.1093/jxb/eraa531)
Supplement: eraa531_suppl_Supplementary_Table_S1 [file eraa531_suppl_supplementary_table_s1.pdf]

Table S1. List of Arabidopsis genes mentioned in the review.

Abbreviation	Gene Number	Gene name	Known or putative function
AOC2	AT3G25770	*Allene oxide cyclase 2*	allene oxide cyclase
CBP60g	AT5G26920	*Calmodulin binding protein 60-like.g*	Transcription factor
DDE2	AT5G42650	*Delayed Dehiscence 2*	allene oxide synthase
DMR6	AT5G24530	*Downy mildew resistant 6*	flavone synthase/SA hydroxylase
EDS1	AT3G48090	*Enhanced Disease Susceptibility 1*	Lipase
EIN3	AT3G20770	*Ethylene-Insensitive 3*	Transcription factor
FRK1	AT2G19190	*Flg22-Induced Receptor-Like Kinase 1*	Receptor kinase
HAK5	AT4G13420	*High Affinity K+ Transporter 5*	K transporter
HSFA1	AT4G17750	*Heat Shock Factor 1a*	Transcription factor
ICS1	AT1G74710	*Isochorismate synthase 1*	Isochorismate synthase/Sa biosynthesis
JAZ1	AT1G19180	*Jasmonate-Zim-Domain Protein 1*	JA signalling
JAZ5	AT1G17380	*Jasmonate-Zim-Domain Protein 5*	JA signalling
JAZ6	AT1G72450	*Jasmonate-Zim-Domain Protein 6*	JA signalling
JAZ7	AT2G34600	*Jasmonate-Zim-Domain Protein 7*	JA signalling
JAZ9	AT1G70700	*Jasmonate-Zim-Domain Protein 9*	JA signalling
LOX1	AT1G55020	*Lipoxygenase 1*	Lipoxygenase
LOX3	AT1G17420	*Lipoxygenase 3*	Lipoxygenase
MYC2	AT1G32640	*Myelocytomatosis oncogenes 2*	transcription factor
MYC3	AT5G46760	*Myelocytomatosis oncogenes 3*	transcription factor
MYC4	AT4G17880	*Myelocytomatosis oncogenes 4*	transcription factor
NAC019	AT1G52890	*NAM/ATAF/CUC 19*	transcription factor
NAC042	AT2G43000	*NAM/ATAF/CUC 42*	transcription factor
NAC053	AT3G10500	*NAM/ATAF/CUC 53*	transcription factor
NAC055	AT3G15500	*NAM/ATAF/CUC 55*	transcription factor
NAC072	AT4G27410	*NAM/ATAF/CUC 72*	transcription factor
NAC092	AT5G39610	*NAM/ATAF/CUC 92*	transcription factor
NPR1	AT1G64280	*Non pathogenesis related 1*	Regulator protein
PAD4	AT3G52430	*Phytoalexin Deficient 4*	Lipase-like
PBS3	AT5G13320	*avrP-phB SUSCEPTIBLE 3*	acyl adenylase
PHR1	AT4G28610	*Phosphate Starvation Response 1*	transcription factor
PDF1.2a	AT5G44420	*Plant Defensin 1.2a*	Antimicrobial peptide
PR1	AT2G14610	*Pathogenesis related 1*	Extracellular protein
PR2	AT3G57260	*Pathogenesis related 2*	β-1,3-Glucanase
PR3	AT3G12500	*Pathogenesis related 3*	Basic Chitinase
PR4	AT3G04720	*Pathogenesis related 4*	Hevein-like
PR5	AT1G75040	*Pathogenesis related 5*	Extracellular protein, thaumatin-like
SARD1	AT1G73805	*Sar Deficient 1*	transcription factor
SIF4	AT1G51820	*Stress Induced Factor 4*	Leucine-rich repeat protein kinase
VSP1	AT5G24780	*Vegetative Storage Protein 1*	Acid phosphatase
WRKY18	AT4G31800	*WRKY DNA-Binding Protein 18*	transcription factor
WRKY22	AT4G01250	*WRKY DNA-Binding Protein 22*	transcription factor
WRKY28	AT4G18170	*WRKY DNA-Binding Protein 28*	transcription factor
WRKY29	AT4G23550	*WRKY DNA-Binding Protein 29*	transcription factor
WRKY30	AT5G24110	*WRKY DNA-Binding Protein 30*	transcription factor
WRKY38	AT5G22570	*WRKY DNA-Binding Protein 38*	transcription factor
WRKY46	AT2G46400	*WRKY DNA-Binding Protein 46*	transcription factor
WRKY50	AT5G26170	*WRKY DNA-Binding Protein 50*	transcription factor
WRKY53	AT4G23810	*WRKY DNA-Binding Protein 53*	transcription factor
WRKY70	AT3G56400	*WRKY DNA-Binding Protein 70*	transcription factor
